# Unveiling Metal Tolerance Mechanisms in *Leersia hexandra* Swartz under Cr/Ni Co-Pollution by Studying Endophytes and Plant Metabolites

**DOI:** 10.3390/metabo14040231

**Published:** 2024-04-18

**Authors:** Mouyixing Chen, Guo Yu, Hui Qiu, Pingping Jiang, Xuemei Zhong, Jie Liu

**Affiliations:** 1 College of Environmental Science and Engineering, Guilin University of Technology, Guilin 541004, China; chenmouyixing@glut.edu.cn (M.C.); qiuhui@glut.edu.cn (H.Q.); liujie@glut.edu.cn (J.L.); 2 Center for Water and Ecology, State Key Joint Laboratory of Environment Simulation and Pollution Control, School of Environment, Tsinghua University, Beijing 100084, China; yuguo@glut.edu.cn; 3 College of Earth Sciences, Guilin University of Technology, Guilin 541004, China; 4 Guangxi Key Laboratory of Exploration for Hidden Metallic Ore Deposits, Guilin 541004, China; 5 Guangxi Key Laboratory of Environmental Pollution Control Theory and Technology, Guilin University of Technology, Guilin 541004, China

**Keywords:** endophytic bacteria, metabolome, nickel, chromium, *Leersia hexandra* Swartz

## Abstract

Heavy metal pollution poses significant environmental challenges, and understanding how plants and endophytic bacteria interact to mitigate these challenges is of utmost importance. In this study, we investigated the roles of endophytic bacteria, particularly *Chryseobacterium* and *Comamonas*, in *Leersia hexandra* Swartz (*L. hexandra*) in response to chromium and nickel co-pollution. Our results demonstrated the remarkable tolerance of *Chryseobacterium* and *Comamonas* to heavy metals, and their potential to become dominant species in the presence of co-pollution. We observed a close relationship between these endophytic bacteria and the significant differences in metabolites, particularly carbohydrates, flavonoids, and amino acids in *L. hexandra*. These findings shed light on the potential of endophytic bacteria to promote the production of aspartic acid and other metabolites in plants as a response to abiotic stressors. Furthermore, our study presents a new direction for plant and bioremediation strategies in heavy metal pollution and enhances our understanding of *L. hexandra*’s mechanisms for heavy metal tolerance.

## 1. Introduction

The electroplating industry is a significant source of heavy metal pollution [1]. The electroplating process discharges large quantities of contaminated wastewater containing heavy metals and other persistent toxic substances [2]. Long-term exposure to electroplating wastewater can have a series of serious health effects on humans, including kidney failure, thyroid dysfunction, insomnia, fatigue, and rheumatoid arthritis, impacting both the circulatory and nervous systems, causing gastrointestinal mucosal irritation, and leading to lung cancer [3]. Wastewater from the electroplating industry also contains multiple highly concentrated heavy metals such as cobalt (Co), copper (Cu), chromium (Cr), mercury (Hg), iron (Fe), nickel (Ni), zinc (Zn), and metalloid arsenic (As) [4,5]. China has approximately 15,000 electroplating companies, generating over 2.7 billion gallons of wastewater annually [6,7]. According to the You et al. [8] research, wastewater from an electroplating plant in Guilin, China, contained Cr and Ni at concentrations of 6.28 and 8.96 mg/L, respectively. Abdel-Shafy et al. [9] collected real electroplating wastewater, in which the metal content exceeded the permissible limits and the concentrations of Ni, Cu, Zn, and Fe were 150, 30, 25, and 2.9 mg/L, respectively. The soil and water pollution generated from this process severely jeopardizes environmental health and safety. Currently, there are various methods available to treat heavy metal pollutants from wastewater, such as ion exchange, membrane filtration, and chemical treatment [10]. However, these methods have some drawbacks, such as high cost, the generation of sludge, high disposal costs of the sludge, and the possibility of leading to secondary pollution [11]. Phytoremediation, on the other hand, is an economical and non-secondary-polluting in situ remediation method [12,13]. Phytoremediation techniques are widely used for heavy metal remediation in soil [14]. However, aquatic plants such as water hyacinth (*Eichhornia crassipes*), water lettuce (*Pistia stratiotes*), and duckweed (*Lemna minor*) also possess phytoremediation potential and can be applied for heavy metal treatment in wastewater [15]. Plants with excellent heavy metal tolerance and accumulation capabilities serve as the primary agents in phytoremediation [16,17]. Nugroho et al. [18] demonstrated the feasibility of phytoremediation of electroplating wastewater by treating Cr and Ni in electroplating wastewater with vetiver as a phytoremediation agent. *Leersia hexandra* Swartz (commonly known as southern cutgrass) is a gramineous plant species with multiple heavy metal tolerances [19]. It can survive in soil containing up to 330 mg Cr/kg and accumulate 1833 mg/kg of Cr in its leaves [20]. Additionally, it can survive in soil containing up to 480 mg Cu/kg, with root and leaf concentrations reaching 755.4 mg/kg and 5501.7 mg/kg of Cu, respectively [21]. In hydroponic experiments with a concentration of 20 mg Ni/L, the roots of *L. hexandra* can absorb 1838.5 mg/kg of Ni, while accumulating 416.2 mg/kg of Ni in the leaves [8,22]. *L. hexandra*, as a chromium hyperaccumulating plant, exhibits strong tolerance not only to Cr but also to Cu and Ni. Additionally, it is a highly prolific aquatic plant with immense potential in the field of phytoremediation. Its versatility allows for widespread application in various environments such as wetlands, farmlands, and riverbanks. Therefore, *L. hexandra* holds great promise as a research subject in the field of phytoremediation technologies. Plants respond to heavy metal stress not only through their own physiological adjustments, but also with a key role played by endophytic bacteria [23].

Endophytic bacteria, which are hosted in plant tissues without harming the plant, are key players in the complex interactions between plants and their environment [24]. These microorganisms play a key role in promoting plant growth, improving resistance and even heavy metal detoxification [25]. For example, *Pseudomonas aeruginosa* CPSB1 possesses the potential to promote plant growth, demonstrates metal tolerance, and has the ability to counteract the harmful effects of metals [26]. *Comamonas* sp. XL8 helps rice seedlings (*Oryza sativa* L.) reduce oxidative stress caused by Cd accumulation, thereby alleviating the toxicity of Cd exposure [27]. Therefore, a thorough understanding of the endophytic microbial communities of plants under different heavy metal stress environments can better facilitate the selection of suitable microorganisms as microbial additives. This, in turn, enhances the plant’s tolerance and remediation capability against heavy metals.

Heavy metals are frequently dangerous to plants, and even a little amount of toxic metal stress can trigger a wide range of physiological responses in plants [28]. Metabolites represent the ultimate products of nearly all cellular and regulatory processes [29]. Consequently, variations in metabolites can unveil the molecular phenotype of an organism. Subtle alterations in an organism’s metabolism during times of adversity can be detected via metabolomics. The metabolism of organic acids, amino acids, flavonoids, and other compounds is changed when there is a heavy metal overload [30,31]. Therefore, gaining a deeper understanding of the changes in metabolites of specific plants under heavy metal stress can lead to a better understanding of the plant’s mechanisms for heavy metal tolerance and detoxification. This knowledge can be more effectively applied in practical plant remediation technologies.

Understanding the intricate interactions between these endophytes and their host plants is essential for optimizing phytoremediation strategies in Cr- and Ni-contaminated environments. In this study, we delve into the complex world of electroplating wastewater pollution, emphasizing the challenges posed by Cr and Ni contamination. We explore innovative approaches for soil remediation, focusing on the potential of phytoremediation aided by endophytic bacteria. By elucidating the mechanisms behind the symbiotic relationship between *L. hexandra* and their associated microorganisms, we aim to enhance our understanding of sustainable solutions for the restoration of contaminated ecosystems. Through comprehensive targeted metabolomics analysis and microbial community amplicon sequencing, we unravel the role of endophytic bacteria in the context of plant-based remediation, shedding light on novel strategies to combat the detrimental effects of industrial pollution.

## 2. Materials and Methods 

### 2.1. Plant Cultures 

*L. hexandra* samples were collected approximately 500 m downstream of a stream near an electroplating factory located in Guilin (north of Guangxi Province). Plants were washed repeatedly with tap water until the roots were free of visible silt, and then rinsed with ultrapure water. The cleaned *L. hexandra* plants were placed in plastic pots with 2 L of 50% Hoagland’s nutrient solution, and incubated in the greenhouse. Hoaglan’s nutrient solution is configured from the general formula of Hoaglan’s nutrient solution produced by Beijing Stream Youth Agricultural Science and Technology Co. (Beijing, China). The main components of Hoagland’s nutrient solution include the following: calcium nitrate 945 mg/L, potassium nitrate 607 mg/L, ammonium phosphate 115 mg/L, magnesium sulfate 493 mg/L, iron salt solution (ferrous sulfate heptahydrate 2.78 g, disodium ethylenediaminetetraacetic acid (EDTA-Na, 3.73 g, distilled water 500 mL) 2.5 mL/L, trace elements solution (potassium iodide 0.83 mg/L, boric acid 6.2 mg/L, manganese sulfate 22.3 mg/L, zinc sulfate 8.6 mg/L, sodium molybdate 0.25 mg/L, copper sulfate 0.025 mg/L, cobalt chloride 0.025 mg/L) 5 mL/L, pH = 6.0. The components of the Hoagland nutrient solution are all sourced from Beijing Stream Youth Agricultural Science and Technology (Beijing, China). The growth conditions were 14 h light (intensity of 7000 Lx), 25 °C/20 °C for day/night cycle, and 65–80% relative humidity. 

### 2.2. Experimental Design

After 7 d of pre-cultivation, relatively promising and similar-growing *L. hexandra* plants were selected and divided into the Cr/Ni treatment group (T group; this includes three sets of biological replicates T1, T2, T3) and control (CK, Hoagland’s solution only; this includes three sets of biological replicates CK1, CK2, CK3) group. Each group will cultivate 25–30 *L. hexandra* columns. According to our previous work [32,33], the experimental pollution concentrations in the T group in this study were set as Ni 40 mg/L and Cr 5 mg/L, and the experiment duration was set at 14 days (after preliminary validation, this stress concentration and duration were determined to be the optimal settings for studying the heavy metal tolerance of *L. hexandra*). At this concentration of heavy metals, *L. hexandra* may exhibit mild toxic reactions but can survive long-term without mortality. These two elements were dissolved in the nutrient solution for cultivation using K_2_Cr_2_O_7_ (Xilong Scientific, Shantou, China) and NiCl_2_·6H_2_O (Xilong Scientific, Shantou, China). After 14 days of treatment, the leaves were collected for metabolomics investigations. They were cut off with scissors and washed in ultrapure water, then promptly frozen in liquid nitrogen, and kept at −80 °C until further examination. To maintain the endophytes within the plant tissue, the roots of *L. hexandra* were disinfected once with 5% sodium hypochlorite (Xilong Scientific, Shantou, China) and then rinsed three times with sterile distilled water. Plant tissue samples were subsequently stored at −80 °C until analysis. All chemicals and reagents were purchased from commercial suppliers and were of analytical grade purity.

### 2.3. Microbial Diversity Analysis Method

The HiPure Soil DNA Kits from Magnen, Guangzhou, China were used to extract microbial DNA in accordance with the manufacturer’s instructions. According to Beckers et al. [5] and Guo et al. [34], the ribosomal RNA gene’s 16S rDNA target area was amplified by PCR using primers 799F (AACMGGATTAGATACCCKG) and 1193R (ACGTCATCCCCACCTTCC). USA-based New England Biolabs provided the relevant PCR reagents. The ABI StepOnePlus Real-Time PCR System (Life Technologies, Foster City, CA, USA) was used to quantify the amplified signals that were extracted from 2% agarose gels and purified using the AxyPrep DNA Gel Extraction Kit (Axygen Biosciences, Union City, CA, USA) in accordance with the manufacturer’s instructions. The normal techniques were followed for pooling purified amplicons for equimolar paired-end sequencing on an Illumina platform. In the NCBI Sequence Read Archive database, the raw reads were entered.

### 2.4. Metabolomics and Statistical Analyses

Prior to being weighed and dissolved in 1.0 mL of extraction solution, the biological samples were freeze-dried under vacuum and crushed into a powder using a grinder (Shanghai Jingxin, TS-24, China). The dissolved material was vortexed three times to speed up the extraction process while it was frozen at 4 °C for the whole night. The vortex oscillator used was a Vortex Genie 2 G560E (Scientific Industries, Bohemia, New York, NY, USA). The material was filtered via a microporous membrane into a sample vial for LC-MS/MS analysis after centrifugation, with the supernatant being aspirated.

The primary components of the data acquisition instrumentation system are tandem mass spectrometry and ultraperformance liquid chromatography (Applied Biosystems 6500 QTRAP, Guangzhou, China). The LC-MS conditions are as follows: the chromatographic column is Xselect HSS T3, 2.5 μm, 2.1 × 150 mm; mobile phase A: 0.1% formic acid/water, mobile phase B: 0.1% formic acid/acetonitrile; column temperature: 50 °C; flow rate: 0.4 mL/min.

To initially demonstrate differences between sample groups, data were examined using the R package and principal component analysis (PCA) and partial least squares–discriminant analysis (PLS-DA). In the projection, the variable significance threshold was set at 1. In addition, the differentially expressed metabolites (DEMs) were screened via univariate analysis using the *t*-test. The DEMs were defined as metabolites having a variable important in projection (VIP) of >1 and *p* < 0.05. 

### 2.5. Statistical Analysis 

SPSS 22.0 was used to conduct a one-way analysis of variance (ANOVA). www.omicshare.com/tools (accessed on 15 December 2023) is a web platform that was used to create all of the visualizations, including heatmaps and volcano plots.

## 3. Results

### 3.1. Data Preprocessing

**Figure 1:**
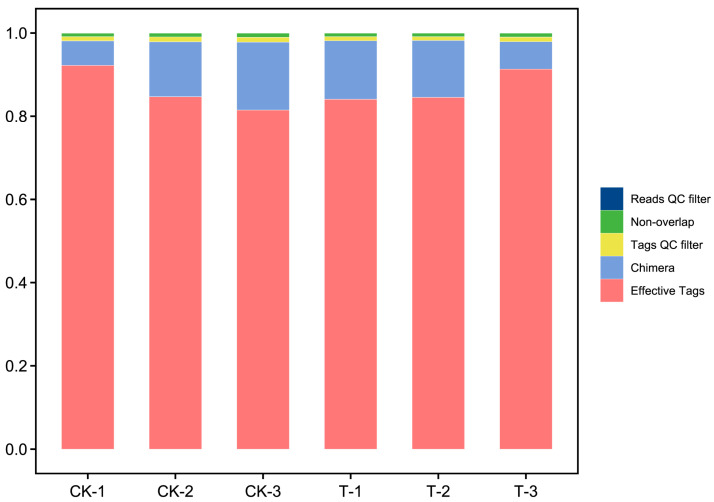
Data preprocessing distribution map.

After acquiring raw reads (127,643 reads per sample on average) through sequencing, the process begins with the initial filtration of low-quality reads. Subsequently, assembly merges paired-end reads into tags, which then undergo further filtering, resulting in clean tags (125,097 clean tags per sample on average). Following this, clustering is conducted based on clean tags, with the removal of any detected chimeric tags during the process, ultimately yielding effective tags (110,287 effective tags per sample on average, and average effective ratio was 86.36%). Data preprocessing statistics and quality control are listed in Table S1, and plot bar graphs which visualize the amount of sequencing data and the filtering analysis effect of the samples are shown in Figure 1.

### 3.2. Alpha Diversity Analysis

**Figure 2:**
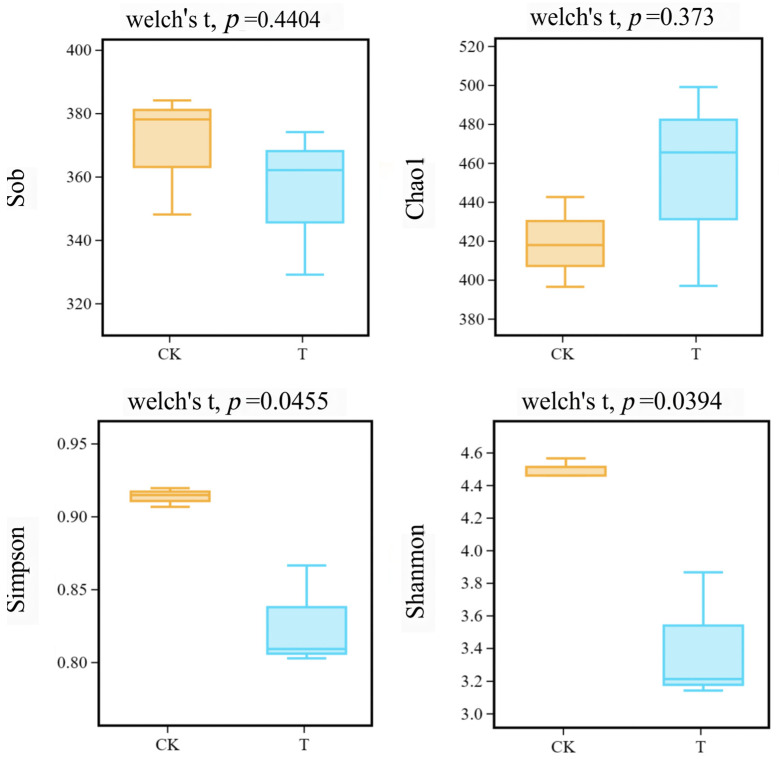
Alpha diversity indices among different groups (yellow box represents the CK group; blue box represents the T group).

Table 1 shows the number of operational taxonomic units (OTUs) per sample, ranging from 329 to 384, and mainstream alpha diversity indices, which include observed species (Sob), Chao1, Shannon, and Simpson. Figure 2 shows that Sobs and Chao1 indices of the CK and T groups have no significant difference (*p* > 0.05), whereas Shannon and Simpson indices of the CK and T groups have a significant difference (*p* < 0.05).

### 3.3. The Taxonomic Composition Analysis

**Figure 3:**
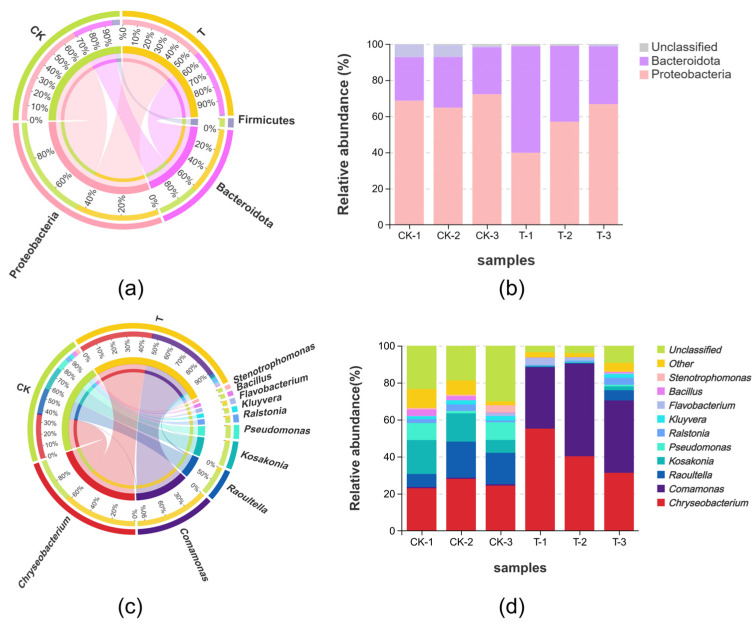
Relative abundance of endophytic bacteria diversity within *L. hexandra*: (**a**) Chord diagram illustrating the phylum-level endophytic bacteria diversity of *L. hexandra* in different groups. (**b**) Diversity of endophytic bacteria at the phylum level; the samples are plotted on the horizontal axis, and the relative proportion of phyla is shown on the vertical axis. (**c**) The genus-level endophytic bacterial diversity of *L. hexandra* in several groups is depicted in a chord diagram. (**d**) Endophytic bacteria diversity at the genus level.

The data analysis showed that *Proteobacteria* was the most dominant phylum in all samples and it had a relative abundance of 68.51% in the CK group and 54.51% in the T group, followed by *Bacteroidota*, with a relative abundance of 26.11% and 44.34% in the CK and T groups, respectively (Figure 3a). At the genus level, the relative abundance of *Raoultella*, *Kosakonia*, and *Pseudomonas* decreased by 12.44%, 12.70%, and 6.25%, respectively, in the CK group compared to the T group, whereas *Chryseobacterium* and *Comamonas* increased by 17.09% and 40.16%, respectively (Figure 3c).

**Figure 4:**
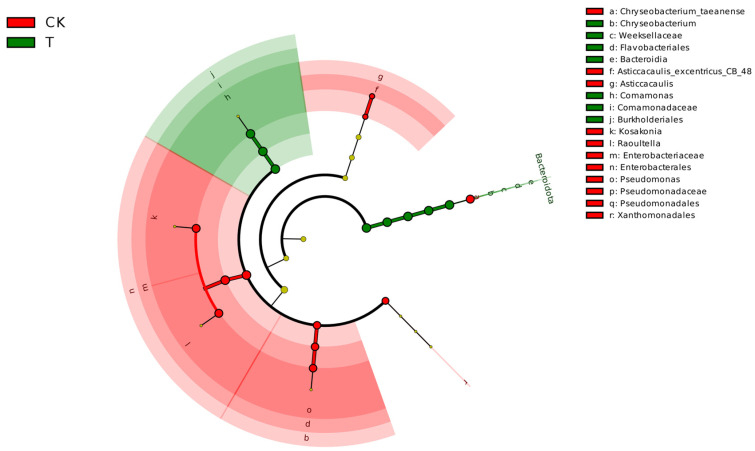
Lefse analysis discrepancy in endophytic bacterial taxonomy of *L. hexandra* between CK group and T group (LDA > 4, *p* < 0.05).

Lefse analysis discrepancy based on datasets of the CK group and T group revealed that *Comamonadaceae*, *Comamonas*, *Burkholdriales*, *Chryseobacterium*, *Bacteroidia*, *Weeksellaceae*, *Flavobacteriales,* and *Bacteroidota* were the main biomarkers in endophytic bacteria of the T group (linear discriminant analysis (LDA) > 4, *p* < 0.05), while *Xanthomonadales*, *Asticcacaulis_excentricus_CB_48*, *Asticcacaulis*, *Pseudomonadaceae*, *Pseudomonadales*, *Pseudomonas*, *Raoultella*, *Kosakonia*, *Chryseobacterium_taeanense*, *Enterobacteriaceae,* and *Enterobacterales* were enriched in the T group (Figure 4). 

### 3.4. 16S-Metabolomic Association Analysis

**Figure 5:**
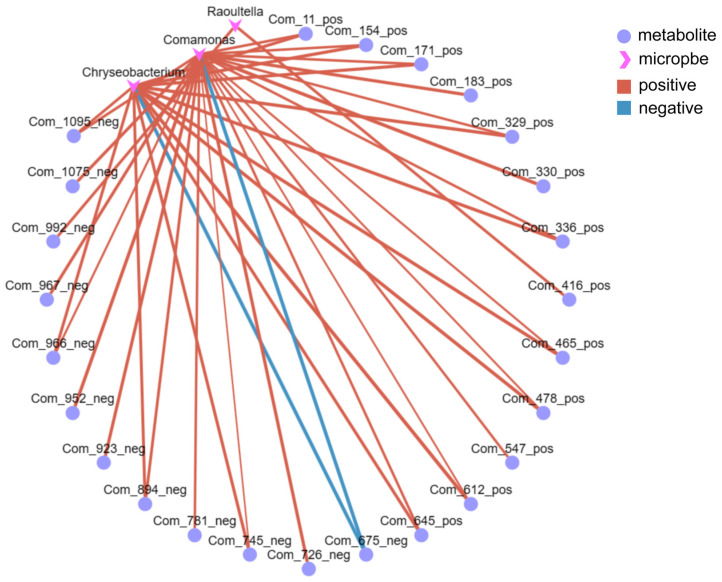
Correlation network diagram. Circles/triangles indicate microorganisms and metabolites, respectively, and the size of the dots indicates the level of connectivity. Positive correlations are shown by red lines, and negative correlations are shown by green lines.

The Pearson correlation coefficient can be used to quantify the mutual relationship between two variables, representing the strength of their co-variation, with values ranging from −1 to +1 (Table S2). The Pearson coefficients of microbial and metabolite abundance were calculated to assess the microbial–metabolite correlation. Based on this, a network graph can be constructed to visually depict the complex interactions among species (Figure 5). Furthermore, the graph’s connectivity (the number of edges attached to nodes) can be used to identify core species within the samples. Core species typically exert significant influence on many other species throughout their overall variations and play a crucial role in group differences, making them potential subjects for subsequent discussion and analysis.

**Figure 6:**
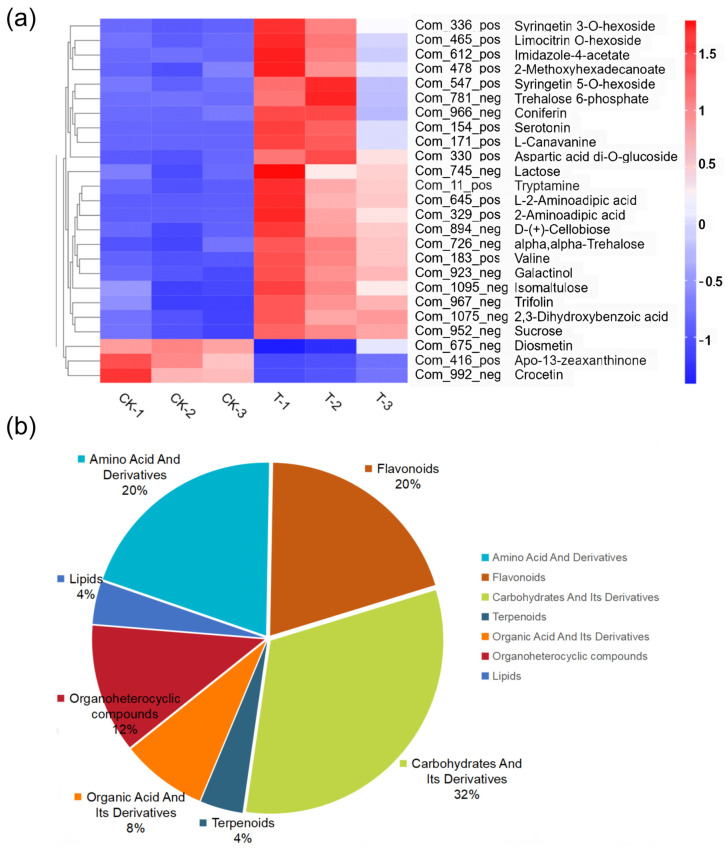
Analysis of DEMs associated with microorganisms between groups (CK vs. T group). (**a**) Heatmap of differentially expressed metabolites (VIP > 1 and *p* < 0.05). (**b**) Pie chart of differential metabolites.

As shown in Figure 5, *Chryseobacterium*, *Comamonas*, and *Raoultella* were the core species, and they were most prominently correlated with metabolites. Notably, *Chryseobacterium* exhibited significant correlations with 14 metabolites, *Comamonas* with 23 metabolites, and *Raoultella* with two metabolites. Figure 6a is the heatmap of differentially expressed metabolites (DEMs) associated with microorganisms. Amino acids, flavonoids, and carbohydrates are the most prominent DEMs significantly associated with microorganisms. Amino acids and flavonoids contribute 20% of total associated metabolites, whereas carbohydrates account for 32% of total associated metabolites (Figure 6b).

## 4. Discussion

We found through examination that endophytic bacteria in *L. hexandra’s* root interact with *L. hexandra*’s metabolites in the leaves under Cr and Ni stress. It has been demonstrated in many studies that endophytic bacteria play important roles when plants are under heavy metal stress, such as assisting in plant detoxification and enhancement of plant growth [35,36,37]. This study employed a combination of comprehensive targeted metabolomics analysis and microbial community amplicon sequencing to investigate the interaction between endophytic bacteria in *L. hexandra’s* root and plant metabolites in the leaves of *L. hexandra* under Cr/Ni stress, and preliminarily elucidated the mechanism of plant tolerance to Cr/Ni composite pollution.

In the present study, according to the alpha diversity analysis, the Shannon and Simpson indices in the T group have decreased compared to the CK group, as shown in Table 1. This indicates a reduction in microbial diversity in the roots of *L. hexandra* in the Cr/Ni-contaminated environment. This can be attributed to the reduction in soil-sensitive bacteria and the proliferation of tolerant bacteria. Some indigenous microorganisms may not be able to adapt to heavy metal pollution and are consequently replaced by microorganisms capable of thriving in such contaminated environments. Some of the notable changes include a decrease in *Raoultella* from 14.47% in the CK group to 2.05% in the T group and a decrease in *Kosakonia* from 12.54% to 0.85%. *Pseudomonas*, as a common metal-tolerant genus, not only assists plants in mitigating the toxicity of heavy metals, but also promotes plant growth [38,39]. However, in this study, we found an abundance of *Pseudomonas* decreasing in amount, from 6.67% in the CK group to 0.42% in the T group (Figure 3d), and we speculate that this is due to the inability of bacteria to adapt to the heavy metal stress environment of Cr/Ni. In contrast, *Chryseobacterium* has increased from 25.11% to 42.19%, and *Comamonas* has surged from 0.70% to 40.87% (Figure 3d). It can be observed that *Comamonas* has transitioned from being virtually absent to becoming a dominant species. Based on these results, we believe that *Chryseobacterium* and *Comamonas* may be able to adapt to environments under heavy metal stress and become dominant bacterial species. Additionally, we speculate that *Chryseobacterium* and *Comamonas* may possess certain mechanisms (such as promoting the secretion of metabolites, enhancing the generation of antioxidant enzymes, etc.) to enhance the heavy metal tolerance, survival ability, and metabolic activity of *L. hexandra* in heavy metal-contaminated environments. According to Wang et al. [40], *Chryseobacterium* has a unique metal tolerance to cadmium. Additionally, according to Majewska et al. [41], *Chryseobacterium* has been reported to limit cadmium mobility within plant tissues, increasing metal bioaccumulation. And it efficiently enhances the bioavailability of nutrients, particularly iron, nitrogen, and phosphorus, in contaminated settings, which helps plants grow better and increases their resistance to heavy metals. Thus, *Chryseobacterium* may play the same role in the complex contamination of Cr/Ni, helping *L. hexandra* to improve heavy metal tolerance and to assist in growth. *Comamonas testosteroni* ZG2 can survive at a Ni(II) concentration of 350 mg/L. It not only induces urease activity, leading to carbonate precipitation but also produces indole-3-acetic acid (IAA) and siderophores. This capability can reduce the bioavailability of Ni while promoting plant growth [42]. Moreover, the co-inoculation of *Comamonas* sp. XL8 and rice seedlings (*Oryza sativa* L.) significantly reduced oxidative stress caused by Cd accumulation [27]. These findings align with our research results, as *Comamonas* exhibits remarkable tolerance to Ni and can even become a dominant bacterial species in the presence of Cr/Ni co-pollution. This underscores its outstanding heavy metal tolerance and suggests its significant potential in the field of bioremediation for heavy metals. In the future, we plan to individually culture and conduct heavy metal tolerance experiments on the *Pseudomonas*, *Chryseobacterium,* and *Comamonas* to validate the hypotheses proposed in this study. Additionally, we will evaluate the impact of these endophytic bacteria on *L. hexandra*’s heavy metal treatment by externally adding them. This will further enhance *L. hexandra*’s ability to handle heavy metals.

Endophytic microbes have been shown to be important members of plant systems in that they promote plant development, particularly in harsh environments, including drought, nitrogen shortage, salt, and metal toxicity [43,44]. Inoculation with endophytic microorganisms has shown the potential to promote plant growth and alleviate stress from polluted and naturally metal-rich soils [45]. In this study, dominant endophytic bacteria (*Chryseobacterium* and *Comamonas*) were closely associated with DEMs present in *L. hexandra*. Through correlation analysis, it was discovered that there is a significant upregulation of most metabolites associated with *Raoultella, Chryseobacterium,* and *Comamonas*, especially *Chryseobacterium* and *Comamonas* (Figure 5). This suggests that these endophytic bacteria may promote the secretion of these metabolites under heavy metal stress. The primary categories of these DEMs in the leaves of *L. hexandra* include carbohydrates, flavonoids, and amino acids (Figure 6b). Carbohydrates are a crucial class of metabolites for plant growth and development, while flavonoids and amino acids play a pivotal role in a plant’s response to abiotic stressors [22,28]. Flavonoids assist plants in balancing excessive reactive oxygen species production and repairing damage caused by them [31]. They are primarily found in the vacuoles of leaf cells and chloroplasts. During periods of stress, the presence of flavonoids in vacuoles aids in detoxifying H_2_O_2_ molecules [46,47]. Further research has also found that under nickel stress, the content of flavonoids in fenugreek inoculated with rhizobia significantly increased, while the content of flavonoids in fenugreek without rhizobia inoculation decreased [48]. This suggests a close connection between flavonoids in plant tissues and microorganisms under nickel stress. Aspartic acid contributes significantly to plants’ ability to withstand abiotic stresses including cold, drought, salt, and heavy metals. It is associated with a number of metabolic processes, such as protein synthesis, nucleotide metabolism, and the TCA cycle [49]. Moreover, in this study, the significant differences observed in aspartic acid and flavonoids were closely related to the presence of endophytic bacteria (Figure 6a), suggesting that *Chryseobacterium* and *Comamonas* may potentially facilitate the production of aspartic acid in the leaves of *L. hexandra* as a response to abiotic stress present in the environment. Furthermore, these endophytic bacteria may have a strong relationship with carbohydrates, indicating that they may have significant potential to influence plant growth and development. In the future, we plan to further validate the results of this study through interventions such as externally adding *Chryseobacterium* and *Comamonas*. Additionally, we aim to analyze the effects of *Chryseobacterium* and *Comamonas* on the metabolites of *L. hexandra* and elucidate how endophytic bacteria enhance the tolerance and accumulation capacity of *L. hexandra* to heavy metals.

These findings offer new insights and avenues for plant and bioremediation strategies in heavy metal pollution. Furthermore, they contribute to a deeper understanding of the mechanisms behind heavy metal tolerance in *L. hexandra*, which can enhance its potential application in future pollution remediation efforts. In future research, we plan to extract these endophytic bacteria for experiments related to heavy metal tolerance and removal, further confirming their capabilities. Simultaneously, we will conduct integrated studies involving endophytic bacteria, plant transcriptomics, rhizospheric metabolites, and other factors to comprehensively investigate the mechanisms of heavy metal tolerance in *L. hexandra*. This will contribute to the advancement of *L. hexandra* in the field of phytoremediation.

## 5. Conclusions 

In conclusion, this study advances our understanding of the intricate interplay between endophytic bacteria and plants in the context of heavy metal pollution. *Chryseobacterium* and *Comamonas*, identified as dominant endophytic bacteria, exhibit exceptional heavy metal tolerance and influence the metabolite profile of *L. hexandra*. Among these, the most significant metabolites include amino acids, flavonoids, and carbohydrates. Through comprehensive targeted metabolomics analysis and microbial community amplicon sequencing, the presence of these endophytic bacteria may significantly influence the secretion of metabolites in *L. hexandra* under non-biological stress, thereby affecting *L. hexandra*’s tolerance to heavy metal exposure. In the future, we plan to further determine the Cr and Ni tolerance capabilities of the two selected microorganisms (*Chryseobacterium* and *Comamonas*) screened and extracted in this study through microbial heavy metal tolerance experiments. Additionally, we aim to assess the impact of these microorganisms on *L. hexandra* under heavy metal stress by externally adding them. This approach introduces new perspectives to practical heavy metal remediation methods.

Our findings open new avenues for future research, where we plan to extract and experimentally confirm the abilities of these endophytic bacteria in heavy metal tolerance and remediation. Integrating plant transcriptomics, rhizospheric metabolite analysis, and other factors will provide a comprehensive understanding of *L. hexandra*’s heavy metal tolerance mechanisms. This holistic approach will facilitate the development and refinement of *L. hexandra*, further enhancing its tolerance, detoxification capacity, and accumulation ability to heavy metals, making it an important plant remediation tool against heavy metal pollution.

## Figures and Tables

**Table 1:** Alpha diversity of endophytic bacteria from *L. hexandra*.

Sample ID	Total Tags	OTUs	α Diversity			
			Sobs	Shannon	Simpson	Chao1
CK-1	116,003	384	384	4.56	0.92	442.40
CK-2	106,264	378	384	4.56	0.92	442.40
CK-3	101,121	348	348	4.46	0.91	396.31
T-1	103,881	329	329	3.21	0.81	396.78
T-2	114,571	362	362	3.14	0.80	498.88
T-3	119,887	374	374	3.86	0.87	465.29

## Data Availability

The raw data supporting the conclusions of this article will be made available by the authors on request.

## Supplementary Materials

The following supporting information can be downloaded at: https://www.mdpi.com/article/10.3390/metabo14040231/s1, Table S1: Data preprocessing statistics and quality control; Table S2: Edge table. The first two columns indicate the metabolite species and the last two columns indicate the correlation between the two, the significance of the correlation.
