# Supplementary material for: Unveiling Metal Tolerance Mechanisms in Leersia hexandra Swartz under Cr/Ni Co-Pollution by Studying Endophytes and Plant Metabolites
Source: Metabolites. 2024 Apr 18;14(4):231. doi: 10.3390/metabo14040231 (PMC11051720; doi:10.3390/metabo14040231)
Supplement: Supplementary file 1 [file metabolites-14-00231-s001.zip › metabolites-2955163-supplementary.pdf]

metabolites

MDPI

SUPPLEMENTARY INFORMATION

Article

Unveiling Metal Tolerance Mechanisms in *Leersia hexandra* Swartz under Cr/Ni Co-Pollution by Studying Endophytes and Plant Metabolites

Mouyixing Chen [1], Guo Yu [2], Hui Qiu [1], Pingping Jiang [3,4,*], Xuemei Zhong [3,4,*] and Jie Liu [1,5]

[1] College of Environmental Science and Engineering, Guilin University of Technology, Guilin 541004, China; chenmouyixing@glut.edu.cn (M.C.); qiuhui@glut.edu.cn (H.Q.); liujie@glut.edu.cn (J.L.)
[2] Center for Water and Ecology, State Key Joint Laboratory of Environment Simulation and Pollution Control, School of Environment, Tsinghua University, Beijing 100084, China; yuguo@glut.edu.cn
[3] College of Earth Sciences, Guilin University of Technology, Guilin 541004, China
[4] Guangxi Key Laboratory of Exploration for Hidden Metallic Ore Deposits, Guilin 541004, China
[5] Guangxi Key Laboratory of Environmental Pollution Control Theory and Technology, Guilin University of Technology, Guilin 541004, China
* Correspondence: jiangpp@glut.edu.cn (P.J.); zxm@glut.edu.cn (X.Z.)

Table S1. Data preprocessing statistics and quality control.

Sample Name	Raw Reads	Clean Reads	Raw Tags	Clean Tags	Chimera	Effective Tags	Effective Ratio (%)
CK-1	125,810	125,739	124,684	123,412	7409	116,003	92.20
CK-2	125,496	125,442	124,311	122,824	16,560	106,264	84.68
CK-3	124,120	124,068	122,850	121,381	20,260	101,121	81.47
T-1	123,575	123,521	122,493	121,280	17,399	103,881	84.06
T-2	135,559	135,501	134,368	133,133	18,562	114,571	84.52
T-3	131,301	131,243	130,001	128,553	8666	119,887	91.31

Table S2. Edge table. The first two columns indicate the metabolite species and the last two columns indicate the correlation between the two, the significance of the correlation.

Compound_ID	Compounds	Phylum	cor	p_value
Com_11_pos	Tryptamine	Proteobacteria	-0.840380859	0.036183995
Com_154_pos	Serotonin	Proteobacteria	-0.869212411	0.024539503
Com_171_pos	L-Canavanine	Proteobacteria	-0.861091461	0.027603213
Com_329_pos	2-Aminoadipic acid	Proteobacteria	-0.873086623	0.023138411
Com_336_pos	Syringetin 3-O-hexoside	Proteobacteria	-0.874866107	0.022508033
Com_465_pos	Limocitrin O-hexoside	Proteobacteria	-0.8984472	0.014945801
Com_478_pos	2-Methoxyhexadecanoate	Proteobacteria	-0.8912878	0.017085115
Com_612_pos	Imidazole-4-acetate	Proteobacteria	-0.924498404	0.008335538
Com_645_pos	L-2-Aminoadipic acid	Proteobacteria	-0.844219541	0.034511122
Com_675_neg	Diosmetin	Proteobacteria	0.841699196	0.035605275
Com_745_neg	Lactose	Proteobacteria	-0.861542328	0.027428637
Com_894_neg	D-(+)-Cellobiose	Proteobacteria	-0.819246115	0.046055158
Com_966_neg	Coniferin	Proteobacteria	-0.849771967	0.032157485
Com_1095_neg	Isomaltulose	Proteobacteria	-0.826714915	0.042439903
Com_11_pos	Tryptamine	Bacteroidota	0.911573017	0.011383277
Com_154_pos	Serotonin	Bacteroidota	0.927503756	0.007693049
Com_171_pos	L-Canavanine	Bacteroidota	0.920410239	0.009249713
Com_183_pos	Valine	Bacteroidota	0.87052496	0.024060433
Com_329_pos	2-Aminoadipic acid	Bacteroidota	0.938298714	0.005593123
Com_330_pos	Aspartic acid di-O-glucoside	Bacteroidota	0.827344088	0.042141654
Com_336_pos	Syringetin 3-O-hexoside	Bacteroidota	0.937638791	0.005712122
Com_465_pos	Limocitrin O-hexoside	Bacteroidota	0.950865596	0.003561975
Com_478_pos	2-Methoxyhexadecanoate	Bacteroidota	0.958100997	0.002596512
Com_547_pos	Syringetin 5-O-hexoside	Bacteroidota	0.842220325	0.035377721
Com_612_pos	Imidazole-4-acetate	Bacteroidota	0.967974902	0.001521988
Com_645_pos	L-2-Aminoadipic acid	Bacteroidota	0.91520818	0.010479667
Com_675_neg	Diosmetin	Bacteroidota	-0.910125971	0.01175304
Com_726_neg	alpha,alpha-Trehalose	Bacteroidota	0.858939195	0.028443801
Com_745_neg	Lactose	Bacteroidota	0.919981343	0.009348299
Com_781_neg	Trehalose 6-phosphate	Bacteroidota	0.835434228	0.038394464
Com_894_neg	D-(+)-Cellobiose	Bacteroidota	0.897632897	0.015182182
Com_923_neg	Galactinol	Bacteroidota	0.860901423	0.027676953
Com_952_neg	Sucrose	Bacteroidota	0.815999287	0.047669605
Com_966_neg	Coniferin	Bacteroidota	0.906444116	0.012719622
Com_967_neg	Trifolin	Bacteroidota	0.8281348	0.041768223
Com_1075_neg	2,3-Dihydroxybenzoic acid	Bacteroidota	0.824044572	0.043716651
Com_1095_neg	Isomaltulose	Bacteroidota	0.882934946	0.019754197
Com_416_pos	Apo-13-zeaxanthinone	Firmicutes	0.846827145	0.03339602
Com_992_neg	Crocetin	Firmicutes	0.814367763	0.04849061
